# ETS proto-oncogene 1 modulates PTP1B expression to participate in high glucose-mediated endothelial inflammation: Ets1 modulates PTP1B expression to participate in inflammation

**DOI:** 10.3724/abbs.2022021

**Published:** 2022-03-02

**Authors:** Lili Jiang, Jincai Liang, Tianhai Wang, Fufen Meng, Wenming Duan

**Affiliations:** 1 Department of Anesthesiology the Third Hospital Affiliated to the Xinjiang Medical University Urumqi 830011 China; 2 Department of Anesthesiology the Fourth People′s Hospital of Qinghai Province Xining 810000 China; 3 Department of Anesthesiology Chengdu BOE Hospital Chengdu 611743 China

**Keywords:** endothelial, ets1, high glucose, inflammation, PTP1B

## Abstract

Hyperglycemia-induced endothelial inflammation participates in the pathogenesis of cardiovascular complications in diabetics. Previous studies showed that protein tyrosine phosphatase 1B (PTP1B) and ETS proto-oncogene 1 (ets1) are involved in hyperglycemia-induced endothelial inflammation. In this study, we hypothesized that ets1 modulates PTP1B expression, thus playing a crucial role in hyperglycemia-induced vascular endothelial inflammation. Our results indicated that high glucose increases monocyte/endothelial adhesion, vascular cell adhesion molecule-1 (VCAM-1) expression and p65 phosphorylation in human umbilical vein endothelial cells (HUVECs). Moreover, high glucose-mediated endothelial inflammation is reversed by PTP1B silencing. In addition, high glucose increases ets1 expression in HUVECs. *Ets1* silencing reverses high glucose-mediated endothelial inflammation. Furthermore, the effect of ets1 overexpression is similar to that of high glucose treatment, which is counteracted by si-PTP1B. The results from ChIP assays indicated that ets1 occupies the PTP1B promoter region. Ets1 overexpression enhances PTP1B promoter activity, which is disappeared after specific binding site mutation. *In vivo* experiments demonstrated that the expressions of VCAM-1, PTP1B, and ets1, as well as the phosphorylation of p65 are augmented in the aorta of diabetic rats. In conclusion, ets1 contributes to hyperglycemia-mediated endothelial inflammation via upregulation of PTP1B expression.

## Introduction

The prevalence of diabetes has increased significantly in the recent years, thus resulting in a great public health problem worldwide [1]. Cardiovascular complication-mediated morbidity and mortality in diabetics are higher than those in non-diabetics [ 2, 3]. Previous studies have shown that high glucose-induced endothelial inflammation plays a crucial role in the occurrence and development of cardiovascular complications in diabetics [ 4– 6].

Hyperglycemia elevates the levels of endothelial adhesion molecules, such as vascular cell adhesion molecule-1 (VCAM-1), via activation of the nuclear factor kappa B (NF-κB) pathway [ 7– 9], thus augmenting the interaction between monocyte and endothelium and leading to endothelial inflammation. Moreover, protein tyrosine phosphatase 1B (PTP1B) was reported to participate in the activation of NF-κB pathway [ 10, 11], thus involving in elevated endothelial cell inflammatory factor levels induced by high glucose. So, PTP1B may be the putative target to improve high glucose-mediated endothelial inflammation.

Ets1, a member of the ETS family of transcription factors, has been reported to participate in hyperglycemia-induced endothelial-to-mesenchymal transition (EMT), thus mediating endothelial injury [12]. Moreover, ets1 has been demonstrated to play an important role in modulation of endothelial adhesion molecule expressions in a model of carotid artery balloon injury [13]. However, the exact mechanism by which ets1 regulates endothelial adhesion molecule expression in hyperglycemia condition is still not well known.

In the present study, we speculated that ets1 participates in high glucose-mediated endothelial inflammation via modulation of PTP1B expression.

## Materials and Methods

### Cell culture and reagents

Human umbilical vein endothelial cells (HUVECs) were purchased from Procell (Wuhan, China). Cells cultured in DMEM (HyClone Laboratories, Logan, USA) supplemented with 5 mM glucose were defined as the control group (con). Cells cultured in DMEM (HyClone Laboratories) supplemented with 25 mM glucose were defined as the high glucose group (HG). Mannitol (20 mM) plus 5 mM glucose was used as an osmotic control. THP-1 cells (Procell) were cultured in RPMI 1640 medium (HyClone Laboratories).

### Western blot analysis

Cells were harvested and lysed using cell lysis buffer (Jiancheng Bio, Nanjing, China). Protein samples were boiled, then separated by 10% SDS-PAGE and transferred to PVDF membranes (Millipore, Billerica, USA). The PVDF membranes were incubated with primary antibodies at 4°C overnight. The primary antibodies used in this study included monoclonal antibodies against VCAM-1 (dilution 1:1000), p-p65 (dilution 1:1000), PTP1B (dilution 1:1000), ets1 (dilution 1:1000) and β-actin (dilution 1:1000) that were all purchased from Abcam (Cambridge, UK). The next day, the membranes were incubated with the corresponding HRP-conjugated secondary antibodies (Abcam). Then, the membranes were detected using the enhanced chemiluminescence (ECL) system (Beyotime Institute of Biotechnology, Shanghai, China).

### Real-time quantitative PCR (qPCR) analysis

Total RNA was extracted using Trizol reagent (Invitrogen, Grand Island, USA). Complementary DNA was transcribed using an RT Reagent kit (TaKaRa Biotech, Dalian, China). qPCR analysis was performed using the AceQ qPCR SYBR Green Master Mix (Yeasen, Shanghai, China) on an ABI7500 Real-Time PCR system (Applied Biosystems, Foster City, USA). The conditions of PCR reaction were as follows: activation of *Taq* DNA polymerase at 95°C for 30 s, then 40 cycles of denaturation at 95°C for 10 s and annealing/extension at 60°C for 30s. The primers used in the present study are listed in Table 1.

**Table:** **Table1** Sequences of primers used for real-time RT-PCR analysis

Gene	Primer sequence (5′→3′)
Human	
*β-actin*	Forward: CGGCTACAGCTTCACCACCAC Reverse: GCCATCTCTTGCTCGAAGTCCAG
*PTP1B*	Forward : CCATATGGAGATGGAAAAGGAGTTCGAG Reverse : CCTAGTCCTCGTGGGAAAGCTCCTTCC
*VCAM-1*	Forward : TTGGCAAAATGGAGCTGTGGT Reverse : ACTGCAAGACCTCAGAGACAA
*ets1*	Forward : TGGAGTCAACCCAGCCTATC Reverse : TCTGCAAGGTGTCTGTCTGC
Rat	
*β-actin*	Forward : CTTCCAGCCTTCCTTCCTGG Reverse : GAGCCACCAATCCACACAGA
*VCAM-1*	Forward : ACTGCACGGTCCCTAATGTG Reverse : CAAGAGCTTTCCCGGTGTCT
*PTP1B*	Forward : ACCCTGTGCGGAAATGCGGG Reverse : GCAGTCAGTCAACCCCGGC
*ets1*	Forward : GAAATGATGTCCCAGGCACT Reverse : CTTTACCCAGGGCACACAGT

### Adhesion of monocytes to HUVECs

HUVECs were incubated in DMEM containing 5 mM glucose or 25 mM glucose for 3 days. After that, 20,000 THP-1 cells were added into different groups of HUVECs and co-incubated at 37°C for 1 h. Then, cells were washed with PBS and observed under a phase-contrast microscope (Olympus, Tokyo, Japan).

### siRNA and plasmid treatments

HUVECs were transfected with si-PTP1B (Biotend, Shanghai, China), si-ets1 (Biotend), si-NC (Biotend), or ets1 plasmids (Biotend) using Lipofectamine 3000 (Invitrogen, Carlsbad, USA) according to the manufacturer’s instructions. The sequences of si-PTP1B that are as follows: si-PTP1B-a, 5′-AUAGGUACAGAGACGUCAGUU-3′, and si-PTP1B-b, 5′-CCAAGAAACUCGAGAGAUC-3′. The sequences of si-ets1 are as follows: si-ets1-a, 5′-CGCUAUACCUCGGAUUACUdTdT-3′, and si-ets1-b, 5′- CCACUAUUAACUCCAAGCAdTdT-3′. The sequence of si-NC is 5′-CAACAAGATGAAGAGCACCAA-3′.

### Immunofluorescence microscopy

For immunocytochemical staining, pretreated HUVECs were grown on glass coverslips in 24-well culture plates. After 24 h of incubation, cells were washed with PBS, fixed for 10 min at room temperature with 4% paraformaldehyde, and permeabilized for 15 min with 0.5% Triton X-100 in PBS. Then cells were blocked with 5% normal serum in PBS for 1 h. Next, the cells were incubated at 4°C with the monoclonal antibodies against ets1 (Abcam) overnight, followed by incubation with a specific fluorescence-conjugated secondary IgG for 1 h in the dark. The cell nuclei were stained by 4′,6-diamidino-2-phenylindole dichloride (DAPI; Yeasen, Shanghai, China). Images were acquired with a fluorescence microscope (Leica, Solms, Germany).

### Chromatin immunoprecipitation (ChIP) assay

ChIP assay was carried out using a SimpleChIP Plus Sonication Chromatin IP kit (Cell Signaling Technology, Beverly, USA) according to the manufacturer’s protocol. Briefly, cells were fixed with 1% formaldehyde and chromatin was sheared with a Microson XL ultrasonic cell disruptor (Misonix, Farmingdale, USA). Then, 10 μl of solution was used as the input. The surplus samples were incubated with anti-ets1 antibody (Abcam) or IgG for 10 h at 4°C. After the immunoprecipitants bound to protein G magnetic beads, the beads were washed and incubated at 65°C for 2 h. After purification, the sequence of DNA fragments was detected by PCR analysis. The primer sequences for *PTP1B* are listed below: forward 5′-CATTATTCAACACACTTCCCA-3′, and reverse 5′-GGACACTTGTGCTATTTTGAG-3′.

### Dual luciferase assay

The PTP1B promoter and the mutant promoter with putative binding site (TTTCCG) delete were amplified and ligated into the pGL3-basic vector (Biotend) to construct the pGL3-PTP1B plasmid. The pGL3-PTP1B plasmid was transfected along with a *Renilla* luciferase vector into HUVECs. The effect of ets1 on PTP1B promoter activity was tested using a dual luciferase assay kit (Promega, Madison, USA) according to the manufacturer’s instructions.

### Rat model of diabetes mellitus

Male Sprague-Dawley rats were obtained from Shanghai SLAC Laboratory Animal Company (Shanghai, China). This study was carried out in accordance with the guide for the Care and Use of Laboratory Animals of Xinjiang Medical University. Rats injected intraperitoneally once with citrate buffer only (0.1 M, pH 4.5) were defined as the control group (con, *n*=10). Rats fed with a high-sugar/high-fat diet for 2 weeks and received a single intraperitoneal injection of streptozotocin (STZ, 50 mg/kg) were defined as the diabetic group (DM, *n*=10). Hyperglycemia was verified by testing the blood glucose through tail-neck blood sampling one week after STZ injection.

### Immunohistochemistry

The aorta tissues of rats were collected and embedded in paraffin and then processed for immunohistochemical analysis. The paraffin sections were incubated with antibodies against VCAM-1 (Abcam), p-p65 (Abcam), PTP1B (Abcam) or ets1 (Abcam) overnight at 4°C. Then, an EnVisionTM Detection Kit (Glostrup, Denmark) was employed to probe signals using diaminobenzidine (DAB) as the enzyme substrate according to the manufacturer’s instructions.

### Statistical analysis

Data are expressed as the mean±SD. The difference between the groups was compared by two-tailed unpaired *t*-test or one-way ANOVAs with GraphPad Prism Version 7.0 (GraphPad Software, San Diego, USA). *P*<0.05 was considered statistically significant.

## Results

### Hyperglycemia-mediated increase of PTP1B, VCAM-1 expressions and p65 phosphorylation in aorta of DM rats

**Figure 1:**
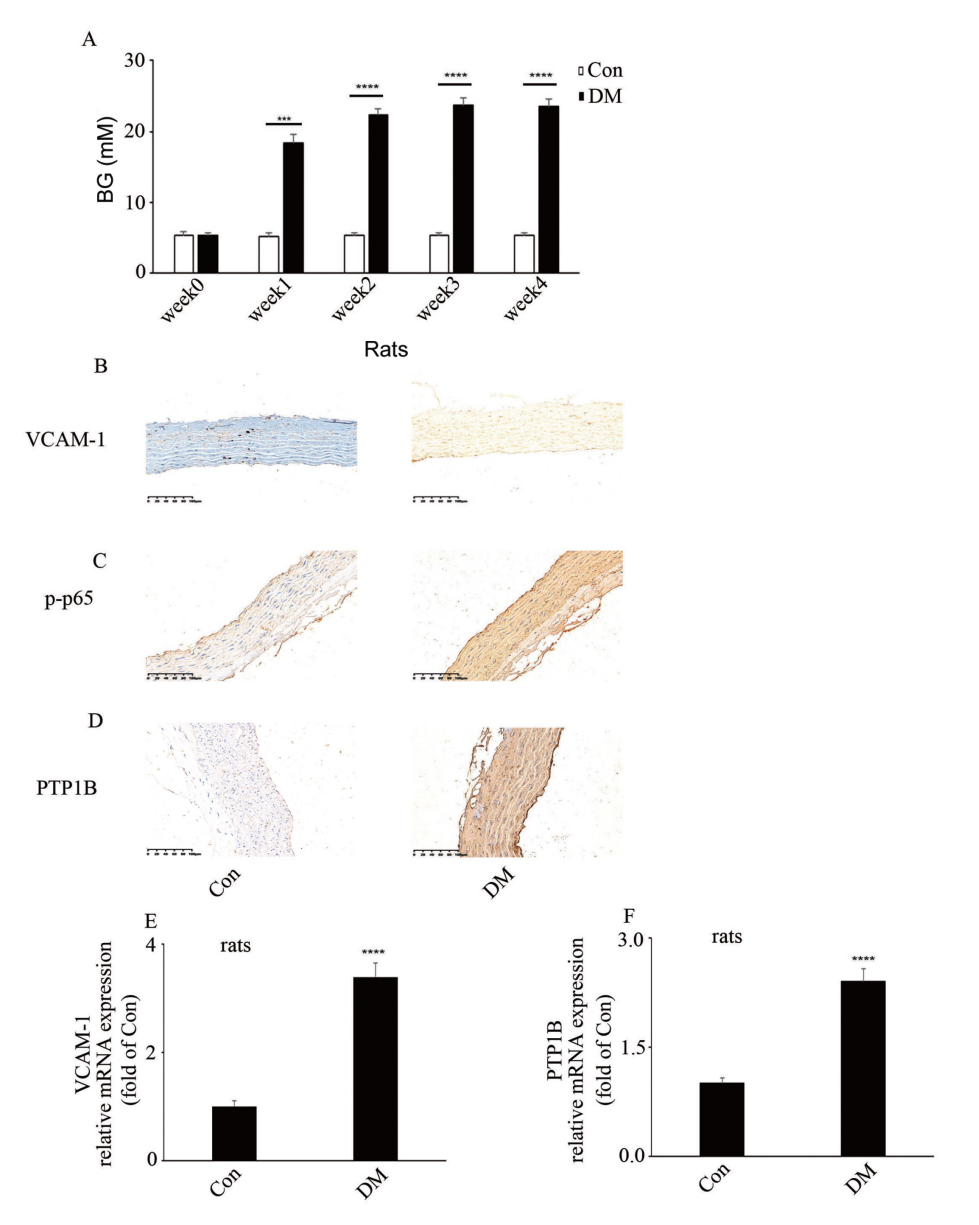
Figure1 Hyperglycemia-mediated increase of PTP1B, VCAM-1 expressions and p65 phosphorylation in aorta of DM rats (A) Blood glucose levels of rats in the control group and in the diabetic group after the induction of diabetes. (B) Immunohistochemical staining of VCAM-1 in aorta biopsy specimen of rats with corresponding treatments. (C) Immunohistochemical staining of p-p65 in aorta biopsy specimen of rats with corresponding treatments. (D) Immunohistochemical staining of PTP1B in aorta biopsy specimen of rats with corresponding treatments. (E) mRNA level of VCAM-1 in aorta of DM rats. (F) mRNA level of PTP1B in aorta of DM rats. ***P<0.001, ****P<0.0001, n=10/group. Scale bar: 100 μm.

Hyperglycemia elevates the levels of endothelial adhesion molecules via activation of the NF-κB pathway [ 7– 9]. Moreover, PTP1B was reported to be involved in the activation of NF-κB pathway [ 10, 11]. To explore the expressions of PTP1B and VCAM-1, as well as the phosphorylation of p65 in DM rats, PTP1B, VCAM-1 expressions and p65 phosphorylation were detected in aortic tissues of DM rats by qPCR and IHC, respectively. The blood glucose (BG) levels in the rats were shown in Figure 1A. The IHC results indicated that PTP1B, VCAM-1 expressions and p65 phosphorylation were augmented in aortic tissues of DM rats compared to those in the control group ( Figure 1B–D). Moreover, the mRNA levels of *PTP1B* and *VCAM-1* were also increased in aortic tissues of DM rats ( Figure 1E,F). These results showed that PTP1B may participate in high glucose-mediated endothelial inflammation.

### High glucose mediates endothelial inflammation and elevates PTP1B level

**Figure 2:**
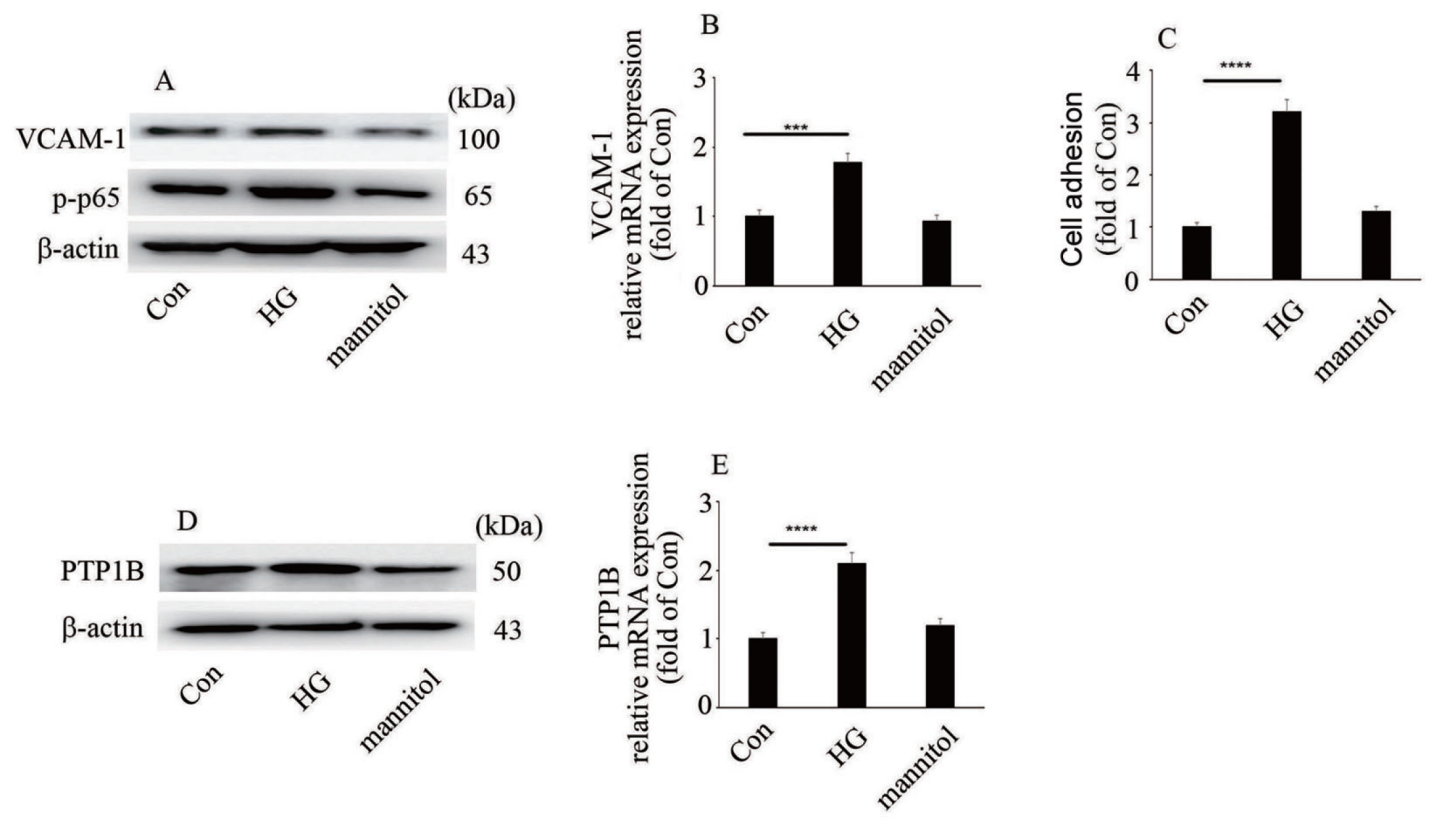
Figure2 High glucose mediates endothelial inflammation and elevates PTP1B level (A) The protein expression of VCAM-1 and p65 phosphorylation in HUVECs with corresponding treatments. (B) The mRNA expression of VCAM-1 in HUVECs with corresponding treatment. (C) Monocyte/endothelial adhesion with corresponding treatments. (D) The protein expression of PTP1B in HUVECs with corresponding treatments. (E) The mRNA expression of PTP1B in HUVECs with corresponding treatments. ***P<0.001, ****P<0.0001.

To explore whether the protein or mRNA expressions of PTP1B, VCAM-1 and phosphorylation of p65 *in vitro* are consistent with those *in vivo*, PTP1B, VCAM-1 expressions and p65 phosphorylation were detected in HUVECs. Our data illustrated that high glucose elevated VCAM-1 level and p65 phosphorylation in HUVECs ( Figure 2A,B). Moreover, high glucose augmented monocyte-endothelial cell adhesion ( Figure 2C). Furthermore, high glucose increased PTP1B protein and mRNA levels in HUVECs ( Figure 2D,E)

### PTP1B downregulation inhibits high glucose-mediated endothelial inflammation

**Figure 3:**
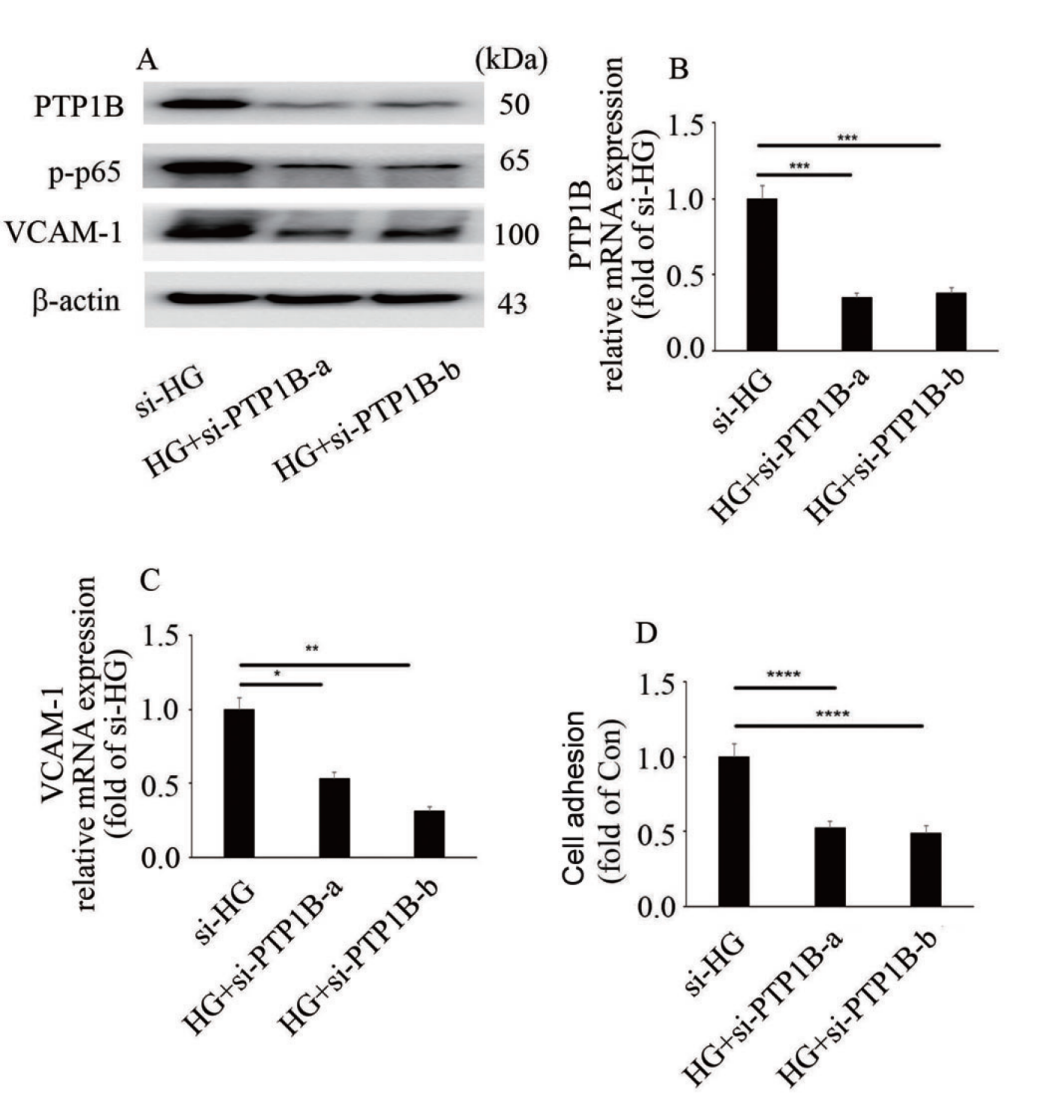
Figure3 PTP1B downregulation inhibits high glucose-mediated endothelial inflammation (A) The protein expressions of PTP1B and VCAM-1, and p65 phosphorylation in HUVECs with corresponding treatments. (B) The mRNA expression of PTP1B in HUVECs with corresponding treatments. (C) The mRNA expression of VCAM-1 in HUVECs with corresponding treatments. (D) Monocyte/endothelial adhesion with corresponding treatments. *P<0.05, **P<0.01, *** P<0.001, ****P<0.0001.

To explore whether high glucose induces endothelial inflammation via upregulation of PTP1B expression, two independent siRNA against PTP1B was used in the present study. The efficiency of si-PTP1B was determined at the protein and mRNA levels ( Figure 3A,B). Our data showed that si-PTP1B reversed high glucose-induced p65 phosphorylation and VCAM-1 expression ( Figure 3A,C), as well as monocyte-endothelial cell adhesion ( Figure 3D). These data showed that high glucose induced VCAM-1 expression and endothelial inflammation via upregulation of PTP1B expression.

### Ets1 downregulation inhibits high glucose-induced endothelial inflammation and PTP1B expression

**Figure 4:**
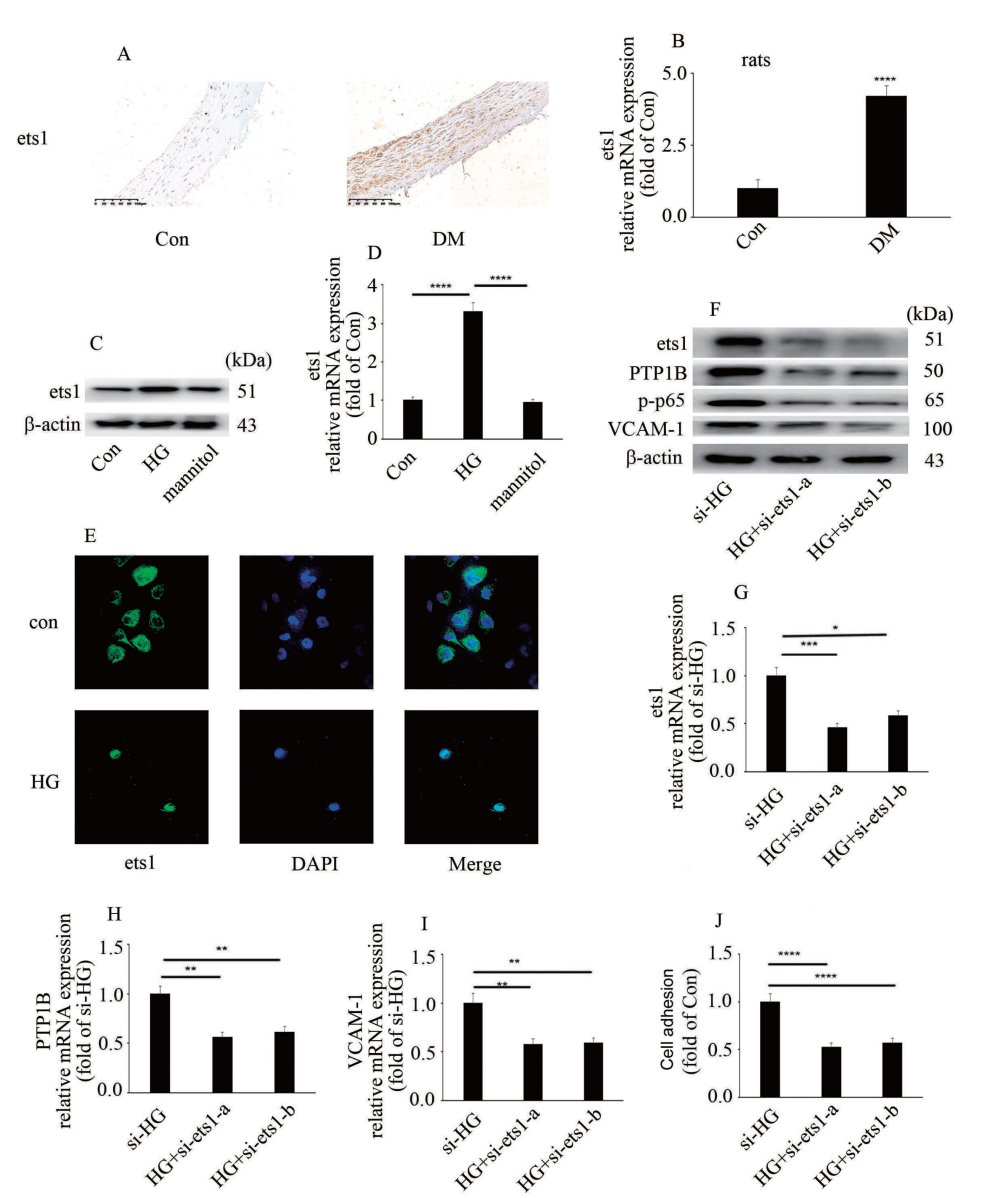
Figure4 Ets1 downregulation inhibits high glucose-induced endothelial inflammation and PTP1B expression (A) Immunohistochemical staining of ets1 in aorta biopsy specimen of rats with corresponding treatments (n=10/group; scale bar: 100 μm). (B) mRNA levels of ets1 in aorta of DM rats. (C) The protein expression of ets1 in HUVECs with corresponding treatments. (D) The mRNA expression of ets1 in HUVECs with corresponding treatments. (E) The distribution of ets1 in HUVECs with corresponding treatments. (F) The protein expressions of ets1, PTP1B, VCAM-1 and p65 phosphorylation in HUVECs with corresponding treatments. (G) The mRNA expression of ets1 in HUVECs with corresponding treatments. (H) The mRNA expression of PTP1B in HUVECs with corresponding treatments. (I) The mRNA expression of VCAM-1 in HUVECs with corresponding treatments. (J) Monocyte/endothelial adhesion with corresponding treatments. *P<0.05, **P<0.01, ***P<0.001, ****P<0.0001, n=10/group.

It has been reported that ets1 participates in the modulation of endothelial inflammation [13]. In the present study, hyperglycemia/high glucose was found to increase ets1 protein and mRNA levels *in vivo* and *in vitro* ( Figure 4A–D). Moreover, treatment with high glucose mediated ets1 nuclear translocation ( Figure 4E). These data indicated that high glucose modulated ets1 expression, as well as ets1 distribution in HUVECs. To explore whether ets1 participates in high glucose-induced PTP1B expression and endothelial inflammation, two independent siRNA against ets1 was used in the present study. The efficiency of si-ets1 was determined at the protein and mRNA levels ( Figure 4F,G). Our data indicated that si-ets1 inhibited high glucose-induced p65 phosphorylation, PTP1B expression and VCAM-1 level in HUVECs ( Figure 4F,H,I). Moreover, ets1 downregulation inhibited high glucose-induced monocyte-endothelial cell adhesion ( Figure 4J).

### Ets1 participates in high glucose-mediated endothelial inflammation via upregulation of PTP1B expression.

**Figure 5:**
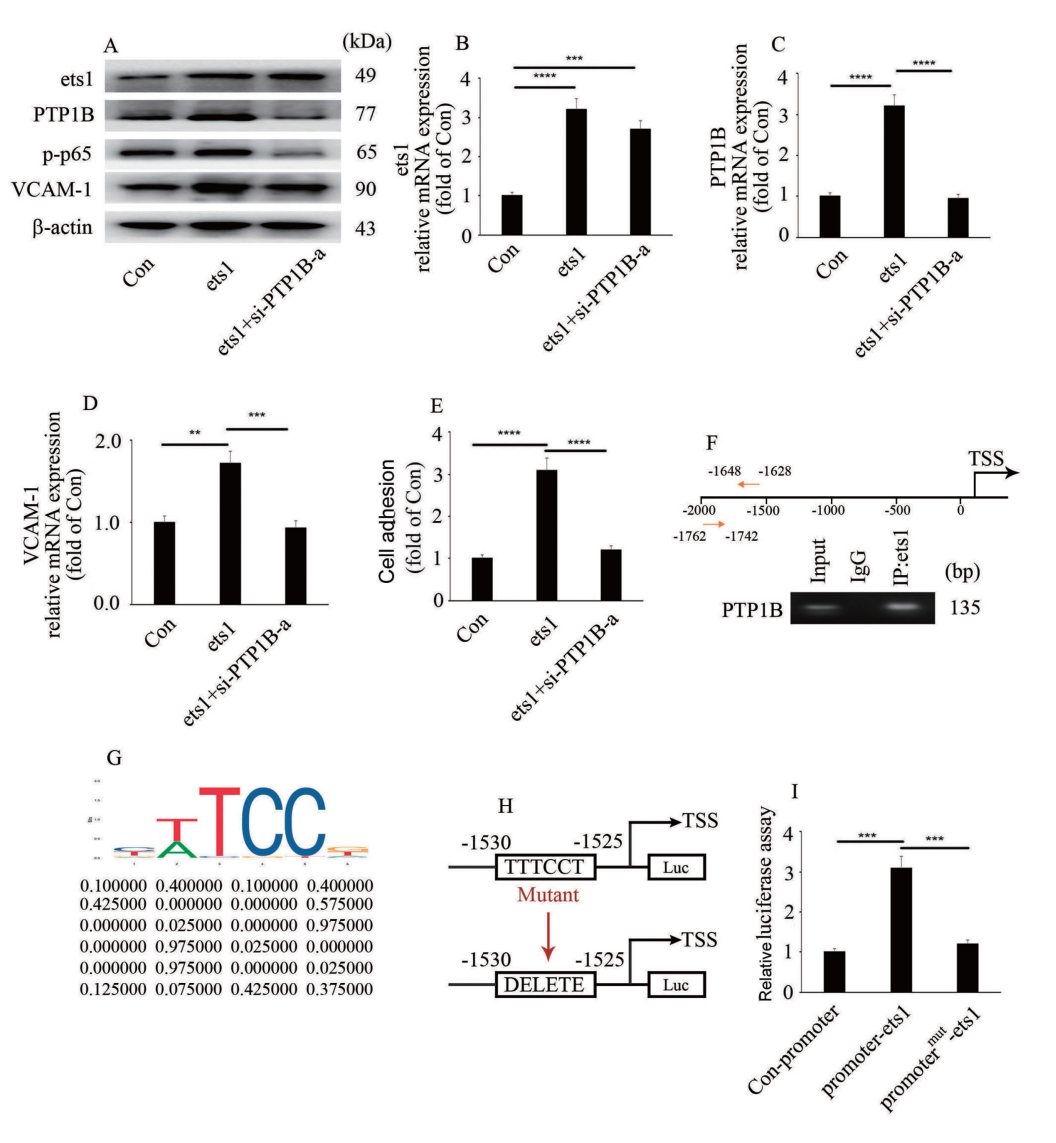
Figure5 Ets1 participates in high glucose-mediated endothelial inflammation via upregulation of PTP1B expression (A) The protein expressions of ets1, PTP1B, VCAM-1 and p65 phosphorylation in HUVECs with corresponding treatments. (B) The mRNA expression of ets1 in HUVECs with corresponding treatments. (C) The mRNA expression of PTP1B in HUVECs with corresponding treatments. (D) The mRNA expression of VCAM-1 in HUVECs with corresponding treatments. (E) Monocyte/endothelial adhesion with corresponding treatments. (F) Ets1 was enriched in the PTP1B promoter region. (G) The putative ets1-binding site in PTP1B. The motif logo and position weight matrix were listed in the up and down panel, respectively. (H,I) PTP1B promoter activity was tested by luciferase reporter assays. **P<0.01, ***P<0.001, ****P<0.0001.

**Figure 6:**
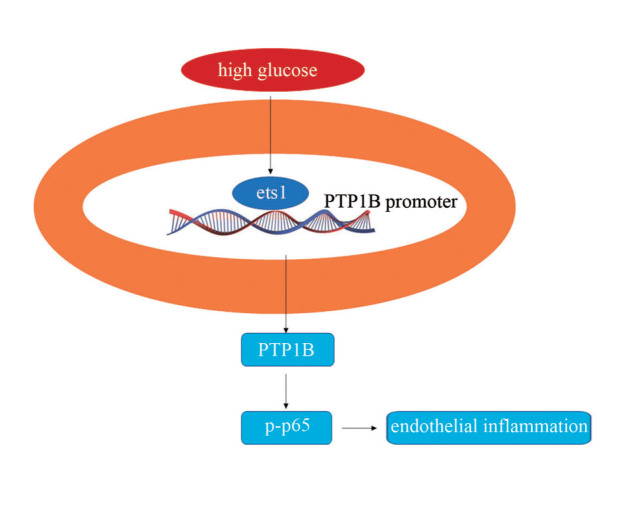
Figure6 Schematic representation of the working model Ets1 contributes to hyperglycemia-mediated endothelial inflammation via upregulation of PTP1B expression.

To explore whether ets1 participates in high glucose-mediated endothelial inflammation via modulation of PTP1B expression, we silenced PTP1B in ets1-overexpressing HUVECs. Our data indicated that the effect of ets1 overexpression was similar to that of high glucose treatment ( Figure 5A–E). Moreover, PTP1B silencing inhibited ets1 overexpression-induced endothelial inflammation ( Figure 5A–E). To determine whether PTP1B is directly transcribed by ets1, the genome-wide distribution of ets1 was detected in this study. ChIP assays indicated that ets1 was enriched in the PTP1B promoter region in HUVECs ( Figure 5F). The putative ets1-binding site is shown in Figure 5G ( http://jaspar.genereg.net/). The binding site (from –1530 bp to –1525 bp) was verified by the results of luciferase assays, which demonstrated that when the sequence of the binding side was mutated, the positive impact of ets1 on the luciferase reporter disappeared ( Figure 5H,I). These data showed that ets1 participated in high glucose-mediated endothelial inflammation via modulation of PTP1B expression ( Figure 6).

## Discussion

The main findings of the present study suggested that high glucose, via augmenting PTP1B levels, induced p65 phosphorylation and VCAM-1 expression, thus participating in endothelial inflammation. Moreover, high glucose elevated ets1 level. Furthermore, ets1 bound with the *PTP1B* promoter region. Mechanistic studies demonstrated that ets1 regulated *PTP1B* transcription in high glucose-treated HUVECs, thus participating in endothelial inflammation.

Hyperglycemia plays an important part in vascular inflammation [14], thus participating in the occurrence and development of cardiovascular complications in diabetics [ 4– 6]. High glucose elevates the levels of endothelial adhesion molecules, thus resulting in the adhesion between monocytes and endothelial cells [ 8, 9]. It was reported that NF-κB signal pathway plays a crucial role in high glucose-induced expressions of endothelial adhesion molecules [ 15, 16]. Moreover, inhibition of NF-κB signal pathway improves high glucose-mediated endothelial inflammation [ 17, 18]. In the present study, we demonstrated that hyperglycemia/high glucose increased VCAM-1 expression ( Figure 1B,E and Figure 2A,B) and monocyte-endothelial adhesion ( Figure 2C). Moreover, hyperglycemia/high glucose elevated p65 phosphorylation ( Figures 1C and 2A). Our data showed that high glucose activated the NF-κB signal pathway, thus inducing endothelial inflammation.

Previous studies have indicated that high glucose-mediated oxidative stress participates in endothelial inflammation [ 19– 21]. Recently, PTP1B has also been reported to take part in endothelial inflammation [11]. It was reported that PTP1B plays an important role in the development of insulin resistance, including diabetes and obesity. Previous studies in PTP1B-knockout mice indicated that PTP1B plays a crucial part in the negative modulation of body mass and insulin sensitivity [ 22, 23]. Mice with brain-specific PTP1B ^–/–^ deficiency showed resistance to diet-induced obesity and elevated insulin sensitivity via central modulation of leptin signal [24]. Moreover, PTP1B is involved in the activation of NF-κB pathway [ 10, 11], thus participating in increased levels of endothelial cell inflammatory factors induced by high glucose. In the present study, high glucose increased PTP1B expression ( Figure 2D,E). Moreover, inhibition of PTP1B expression attenuated high glucose-induced VCAM-1 expression and p65 phosphorylation ( Figure 3A–C), as well as monocyte/endothelial interaction ( Figure 3D). Our data indicated that PTP1B may be a potential therapeutic target to improve high glucose-mediated endothelial inflammation.

The ETS protein family is a group of transcription factors which play crucial roles in the modulation of cell proliferation, differentiation, and survival. Moreover, ETS transcription factors participate in the modulation of specific gene expressions, which are involved in vascular homeostasis, development, and angiogenesis [25]. At present, no ETS-specific transcription factors have been identified. Ets1, as a member of the ETS family of transcription factors, is involved in hyperglycemia-mediated endothelial-to-mesenchymal transition, thus mediating endothelial injury [12]. Moreover, ets1 was demonstrated to play an important role in the modulation of expressions of endothelial adhesion molecules in a model of carotid artery balloon injury [13]. In the present study, ets1 expression was found to be increased in aorta of DM rats and high glucose-treated HUVECs ( Figure 4A–D). Moreover, ets1 silencing improved high glucose-induced PTP1B expression and endothelial inflammation ( Figure 4F–J). In addition, ets1 overexpression-induced PTP1B expression and endothelial inflammation were reversed by *PTP1B* silencing ( Figure 5A–E). Furthermore, ets1 was verified to bind with the *PTP1B* promoter region ( Figure 5F). These data showed that ets1 participates in high glucose-mediated endothelial inflammation via modulation of PTP1B expression.

Nevertheless, the present study has some limitations. First, only HUVECs were used to establish an *in vitro* model, other primary endothelial cells should be used to confirm the results of this study in the future. Second, the mechanistic studies were mainly carried out *in vitro*, and these results should also be confirmed *in vivo*. Third, *in vivo* intervention experiments should be performed in the future to verify our *in vitro* results of this study.

In summary, our data indicated that ets1, PTP1B and VCAM-1 expressions, as well as p65 phosphorylation are increased in HUVECs as well as in aortic tissues of DM rats. We also demonstrated that high glucose induces endothelial inflammation via upregulation of PTP1B expression in hyperglycemic HUVECs. Moreover, high glucose elevates ets1 level ( Figure 6). Furthermore, ets1 participates in high glucose-mediated endothelial inflammation via increasing PTP1B expression
